# Textile Radio-Frequency Active Devices and Systems: Wireless Communication and Energy Harvesting

**DOI:** 10.34133/research.1101

**Published:** 2026-03-10

**Authors:** Wenzhe Song, Hao Chen, Zhenghao Kou, Zehui Chen, Jianing Li, Tian Liu, Xingce Fan, Weibing Lu

**Affiliations:** ^1^ State Key Laboratory of Millimeter Waves, School of Information Science and Engineering, Southeast University, Nanjing 210096, China.; ^2^ Center for Flexible RF Technology, Frontiers Science Center for Mobile Information Communication and Security, Southeast University, Nanjing 210096, China.; ^3^ Key Laboratory of Quantum Materials and Devices of the Ministry of Education, School of Physics, Southeast University, Nanjing 211189, China.; ^4^ Donghua University, Shanghai 201620, China.

## Abstract

The integration of wearable technology and smart textiles has substantially advanced the development of radio-frequency (RF) electronics embedded in textile substrates, opening new opportunities across health monitoring, environmental sensing, and wireless communication. Despite their established performance, conventional rigid RF systems face inherent limitations in conformability and seamless integration within wearable platforms. This review comprehensively summarizes recent progress in textile-based RF active devices, encompassing reconfigurable antennas, tunable metasurfaces, and multifunctional RF systems. We emphasize the transition from isolated components to fully integrated, intelligent platforms capable of energy harvesting, low-power communication, and distributed sensing while proposing solution strategies and future development directions for enriched and systematic performance. Critical technical challenges such as high-precision fabrication, tunable RF response, and architecture design for complex systems are thoroughly discussed. Ultimately, this review outlines promising pathways toward autonomous, adaptive, and intelligently networked textile RF systems, highlighting the convergence of wireless functionality and system-level co-design for the next generation of wearable electronics.

## Introduction

Advances in material development and manufacturing processes have accelerated the progress of flexible electronics [1–5], giving rise to smart textiles [6–8]. This shift enables the transition from conventional plastic-film substrates to textile-based platforms, facilitating direct integration of electronic functions into fibers and textiles. Such innovations have created unprecedented opportunities for environmental sensing [9–12], health monitoring [13–18], and wireless communication [19–21]. Modern applications require devices that are lightweight, flexible, and seamlessly integrated into daily clothing, enabling continuous monitoring and interaction without compromising user comfort. These needs have motivated the emergence of textile-integrated radio-frequency (RF) technologies, which embed antennas [20–23], metasurfaces [19,24,25], and sensing functionalities [17] directly within fabrics. Such integration allows for distributed, unobtrusive, and intelligent wearable systems [14,18] capable of real-time communication, environmental awareness, and energy management, laying the foundation for next-generation smart textiles.

**Fig. 1.**
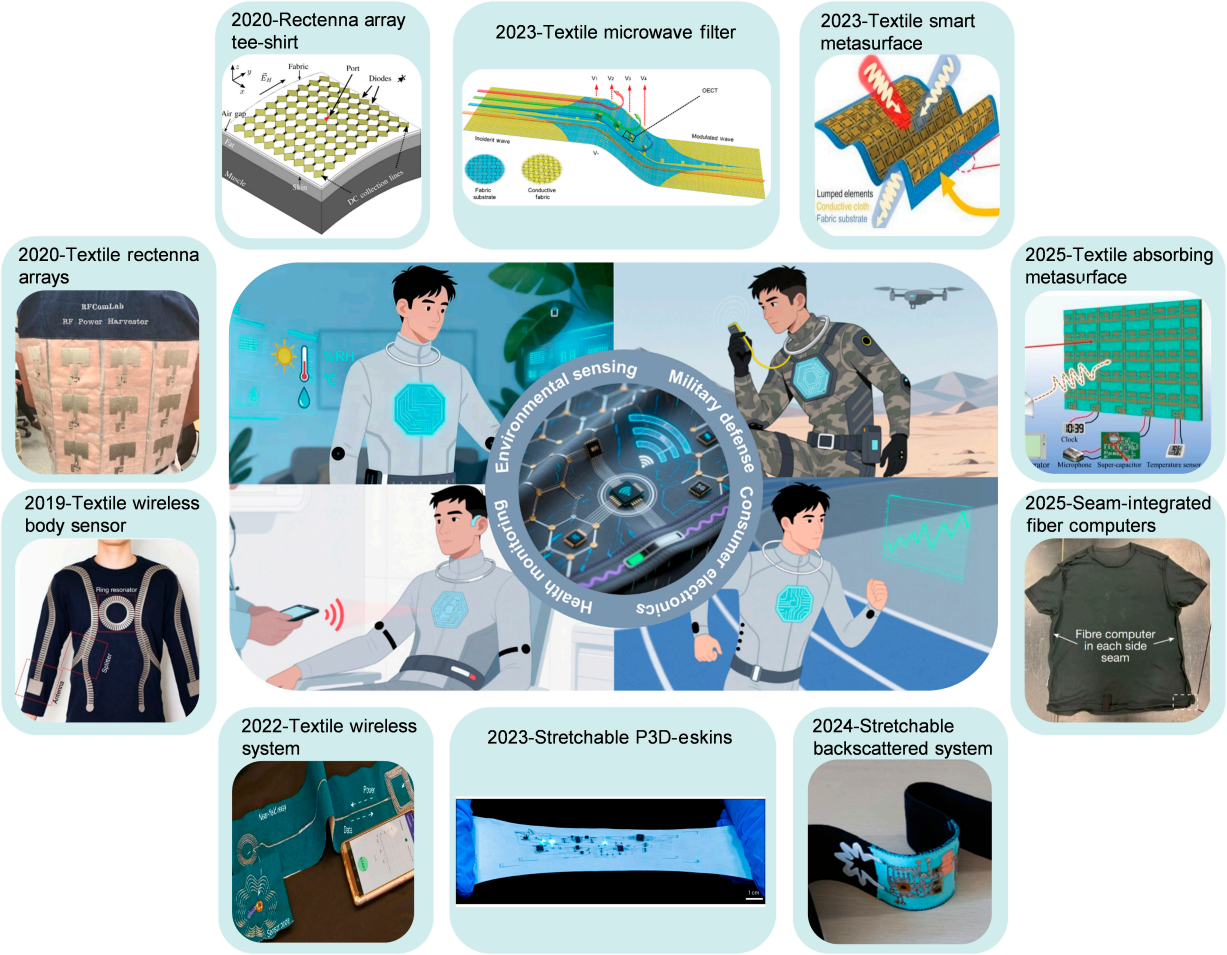
Overview of recent advances in applications for textile-based active devices and systems following chronological order. Textile wireless body sensor: Reproduced with permission from [30]. Copyright 2019, Springer Nature. Textile rectenna arrays: Reproduced with permission from [32]. Copyright 2020, IEEE. Rectenna array tee-shirt: Reproduced with permission from [89]. Copyright 2020, IEEE. Textile wireless system: Reproduced with permission from [16]. Copyright 2022, Springer Nature. Textile microwave filter: Reproduced with permission from [105]. Copyright 2023, John Wiley & Sons Inc. Textile smart metasurface: Reproduced with permission from [10]. Copyright 2023, John Wiley & Sons Inc. Stretchable P3D-eskins: Reproduced with permission from [14]. Copyright 2024, Springer Nature. Stretchable backscattered system: Reproduced with permission from [18]. Copyright 2024, John Wiley & Sons Inc. Textile absorbing metasurface: Reproduced with permission from [25]. Copyright 2025, IEEE. Seam-integrated fiber computer: Reproduced with permission from [9]. Copyright 2025, Springer Nature.

Despite their technological maturity and proven performance, conventional RF devices [26,27] face challenges when deployed in wearable platforms. Rigid form factors, bulky packaging, and designs optimized for conventional applications can limit miniaturization, conformability, and seamless integration within garments or fabrics. Moreover, traditional RF circuits are often designed for isolated or single-function operation, making system-level coordination, multi-device integration, and long-term autonomous operation difficult to achieve in wearable contexts. These considerations highlight the need to rethink materials, RF design, and architectures specifically for textile-based applications. Textile-based (or called fabric-based) RF active electronics offer a transformative approach to overcoming the constraints of conventional devices (Fig. 1). By embedding conductive fibers and electronic components directly into fabrics, researchers have constructed key RF components. For instance, textile antennas have evolved from simple patch designs to actively reconfigurable systems integrating RF switches or varactor diodes for dynamic frequency or pattern control. Similarly, smart metasurfaces have been realized by incorporating diodes or organic electrochemical transistors (OECTs) onto fabric, enabling adaptive electromagnetic (EM) responses. The progression of textile-based RF systems has evolved from bulky, externally attached printed circuit board (PCB) modules to more conformal stitched-on flexible printed circuit board (FPCB) designs [10,17], to chip-embedded fiber [14,28], and finally to revolutionary monolithic “fiber computers” [9] where computation and communication are embedded within the yarn itself, vividly demonstrating the fundamental shift toward achieving inherent flexibility and conformability while preserving RF functionality. Such platforms facilitate seamless multi-device integration [15,29], energy harvesting [30–32], and distributed sensing [16,22], enabling more intelligent and autonomous wearable systems. Nevertheless, realizing these advantages requires addressing critical challenges, including high-precision patterning on fibrous substrates [33–36], reliable interconnects between fiber and lumped elements (resistors, capacitors, inductors, etc.), and active chips [diodes, microcontroller units (MCUs), switches, etc.] under repeated deformation [37–40], stable multi-source energy management [41–43], and system-level coordination of multifunctional components. Success in these areas demands interdisciplinary efforts spanning materials science, artificial intelligence (AI)-accelerated EM design [44–47], advanced manufacturing [48–50], and system integration, highlighting both the opportunities and complexities of textile-based RF technologies.

**Fig. 2.**
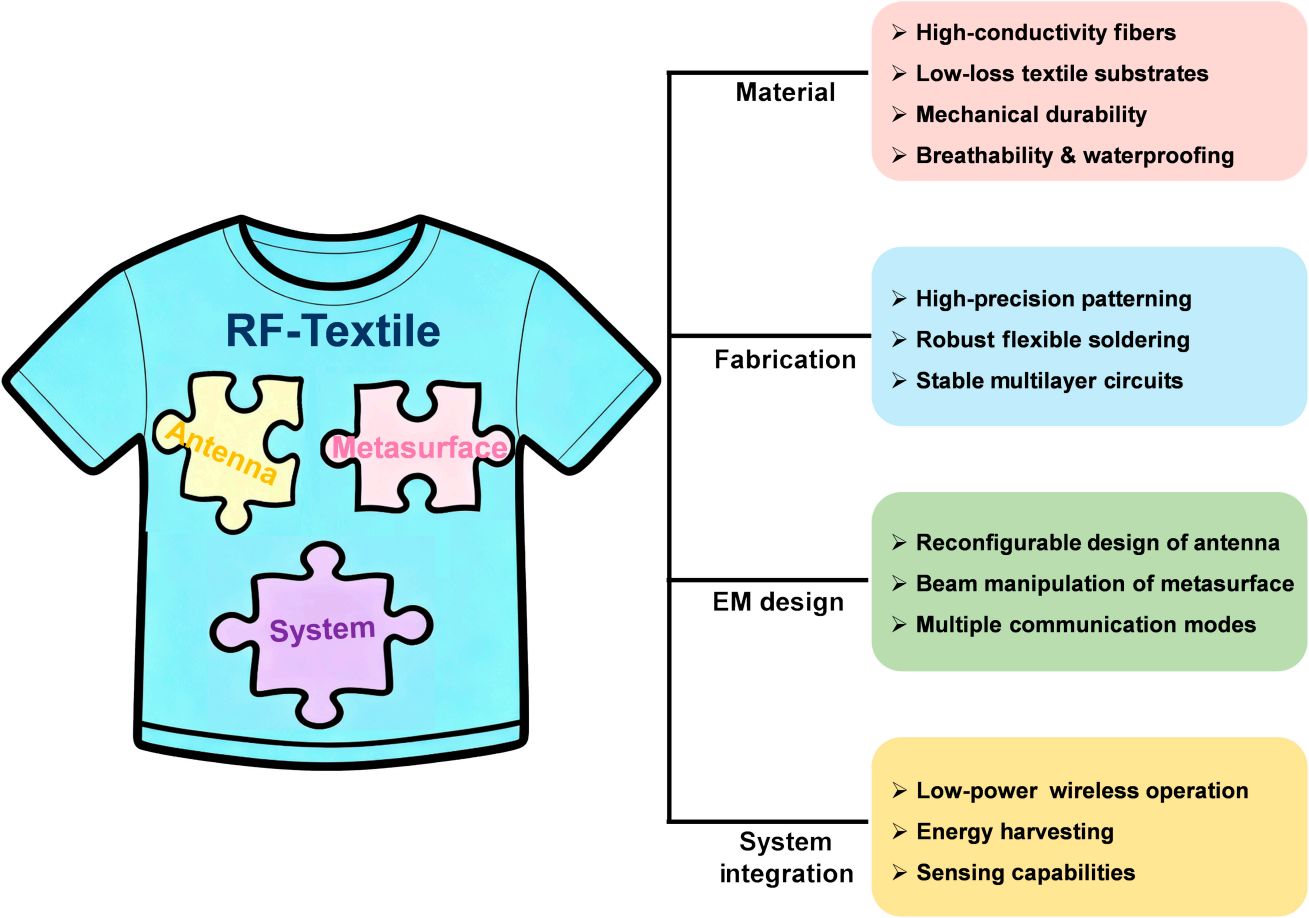
Key challenges in the components of textile-integrated RF system.

While numerous reviews [8,51–54] have been dedicated to flexible and textile electronics, most have primarily focused on flexible material fabrication [55,56], manufacturing techniques [57,58], and multifunctional applications [59–61]. What remains notably underexplored is the distinction between conventional electronics and RF electronics in the context of wireless smart textiles, as opposed to conventional low-frequency or digital textile electronics. A systematic analysis dedicated to the functional taxonomy of textile RF active devices and the architecture evolution of textile RF systems is notably lacking. Crucially, the difficulties in achieving and maintaining RF performance represent a unique set of hurdles that must be distinguished from those of general textile electronics. These include realizing high-frequency circuit patterning, ensuring reliable integration of active chips, maintaining impedance stability under deformation, and preserving radiation efficiency on lossy fabrics. To address this gap, this review systematically examines the textile RF domain, positioning it as a critical enabler for next-generation wireless smart textiles. Specifically, this review summarizes recent advances in textile antennas with embedded electronics, smart metasurfaces, and wearable RF systems. We first discuss the design and performance of textile-integrated active antennas and metasurfaces as fundamental building blocks and then examine strategies for multi-device, multifunctional system integration, highlighting the evolution toward highly flexible, wearable platforms (Fig. 2). In the last section, we explore emerging concepts for fully autonomous and intelligent textile-based RF systems, including energy self-sufficiency, low-power communication, and environment-aware functionalities, thereby establishing a coherent framework that tracks the progression from “textiles as passive carriers” to “textiles as fully integrated systems”. By connecting device-level innovations to system-level architectures and practical applications, this review provides a comprehensive perspective on the current state, challenges, and future directions of textile-based RF electronics, bridging fundamental research with potential societal impact.

## Textile Antenna with Embedded Electronics

Antennas, serving as the critical bridge for transmitting and receiving EM waves, play a pivotal role in wireless communication systems. Compared to conventional passive antennas [62,63], conventional antennas with embedded electronics (active electronic components, such as varactor diodes, positive-intrinsic-negative (PIN) diodes, and rectifier diodes) can realize more complex functions like the reconfigurability of frequency [64–66], polarization [67–69], and radiation pattern [70,71], and energy harvesting [72–76]. Textile antennas with embedded electronics offer a decisive advantage over rigid antennas by not only delivering functional benefits like adaptive tuning, signal enhancement, and energy autonomy but also providing inherent softness, lightweight form factor, thinness, and breathability. These capabilities make them highly suitable for biomedical monitoring, tactical communications, sports wearables, and consumer Internet of Things (IoT) applications.

The development of textile antennas with embedded electronics presents significantly greater challenges compared to their passive counterparts that merely require conductive pattern integration. Fabrication of textile antennas with embedded electronics faces 3 critical technical hurdles: ensuring stable compatibility between textile-compatible conductive patterning processes and active component integration, and achieving effective tunable circuit designs or high-efficiency rectifying circuits for active control purposes. Consequently, despite the remarkable progress achieved in recent years, textile antennas with embedded electronics remain under active development, and further efforts are needed to address the key integration and performance challenges. The following discussion primarily categorizes textile antennas with embedded electronics into textile reconfigurable antennas and textile rectifying antennas for detailed elaboration.

### Textile reconfigurable antenna

**Fig. 3.**
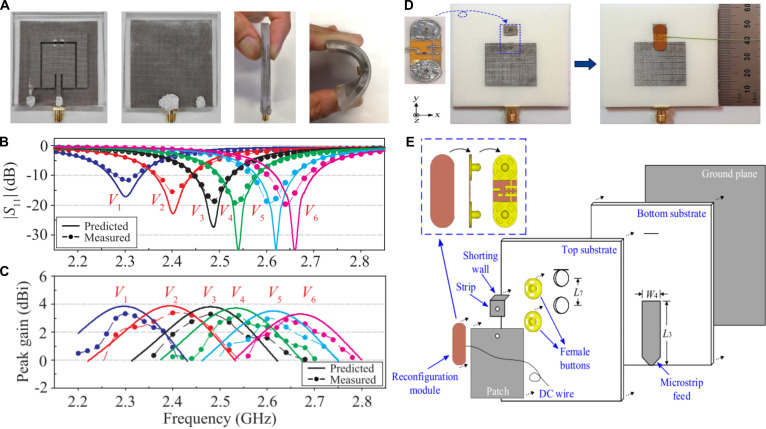
Textile frequency-reconfigurable antennas. (A) Photograph of a flexible polymer-embedded conductive fabric tunable antenna at the front, back, sides, and curved states. (B) S11 parameters and realized gains of the antenna in free space, with different voltages. The capacitance variation of the varactor diode under different applied voltages enables the antenna’s resonant frequency to shift from 2.3 to 2.65 GHz, while (C) the peak gain changes between 2.9 and 3.3 dBi. Reproduced with permission from [23]. Copyright 2018, IEEE. (D) Photograph of a textile antenna with the reconfiguration module at the front and back. (E) Schematic representation of the structure composition of the textile antenna. Reproduced with permission from [21]. Copyright 2021, IEEE.

In terms of functional reconfiguration dimensions [77], textile reconfigurable antennas can be primarily classified into frequency reconfiguration [20,21,23,78], polarization reconfiguration, radiation pattern reconfiguration [79–82], and hybrid-mode reconfiguration [83,84]. Among these, operating frequency is the most critical parameter in antenna design, as it plays a central role in defining the physical dimensions and applicable scenarios of the antenna. Conventional passive antennas, constrained by their fixed frequency response characteristics, may not fully meet the stringent requirements of contemporary wireless communication systems. Frequency-reconfigurable antennas have attracted significant research interest due to their ability to dynamically adapt to different communication frequency bands. They are typically achieved by employing RF switches or varactor diodes to modulate the antenna’s resonant length or reactance, and utilizing mechanical or electrical tuning methods to alter the dielectric constant of antennas [85]. These mechanisms enable the antenna to exhibit either discrete or continuous frequency tunability within a specific bandwidth. In recent years, textile frequency-reconfigurable antennas have shifted the primary research focus to reconfigurable designs and composite material designs that combine conductive textiles with polymer substrates or integrate copper foils with fabric bases, thereby maintaining high dielectric constants in the substrate materials while achieving high conductivity in the radiating elements [84,86]. To achieve robust integration of textiles and electronic components, Simorangkir et al. [23] proposed using conductive fabric as the radiating element and ground plane of the antenna, co-integrating them with active components on a polydimethylsiloxane (PDMS)-based substrate, followed by PDMS encapsulation to fabricate a frequency-reconfigurable antenna (Fig. 3A). As depicted in Fig. 3B and C, by applying bias voltage to varactor diodes via a Bias Tee, the antenna achieved frequency tunability from 2.3 to 2.68 GHz. Experimental results demonstrated that this robust fabrication method maintained excellent impedance matching even when the antenna was bent on different parts of the human body or after washing. In another study focusing on wearability comfort and multi-band operation, Tahir and Javed [78] developed a frequency-reconfigurable antenna using a flexible denim substrate. By incorporating a single PIN diode controlled via a simple bias circuit, the antenna’s electrical length was altered, enabling dynamic switching between 2.45 and 5 GHz. The reliability of the antenna was further validated under on-body conditions. Nevertheless, these antenna designs that directly integrate varactor or PIN diodes still face practical challenges, such as discontinuous frequency-tuning capability and a restricted frequency-tuning range. To achieve more flexible control, Fumeaux’s research group [20] proposed an innovative modular design paradigm for textile antennas. Their approach involved developing a dedicated coplanar reconfiguration module integrating varactor diodes, bias circuits, and matching networks using standard FPCB fabrication techniques, establishing reliable interconnections between the rigid module and flexible textile substrate through snap-button coupling mechanisms. This architecture demonstrated significant performance advantages when applying controlled reverse bias voltage to strategically placed varactor diodes along the patch edge. The textile antenna achieved discrete tuning ranges of 32.8% (4.5 GHz) and 8.8% (5.8 GHz) while maintaining robust mechanical integration between electronic components and textile. The study effectively resolved the critical trade-off between frequency-tuning capability governed by electronic components and structural flexibility determined by textile substrates in wearable antenna design. To further expand the frequency-tuning range, the above research team adopted a similar fabrication strategy to develop an ultra-wideband tunable textile antenna [21]. As illustrated in Fig. 3D and E, the antenna’s radiating patch, microstrip feedline, ground plane, and shorting plane were constructed using silver-coated nylon rip-stop fabric, while the dual-layer dielectric substrate was made from Cumming Microwave PF4 foam. By incorporating a coplanar reconfiguration module into a planar inverted-F antenna (PIFA) structure, the tuning circuit allowed varactor capacitance values to be adjusted from 0.1 to 5 pF via bias voltage control to achieve a continuous transition between quarter-wave PIFA mode and half-wave patch mode, ultimately realizing an impressive 70% frequency-tuning range from 1.82 to 3.79 GHz.

The polarization characteristics of EM waves describe the oscillation pattern of the electric field vector along the propagation direction. Optimal signal transmission efficiency is achieved when the polarization states of the transmitting and receiving antennas are matched. Traditional polarization control primarily relies on manipulating the surface current distribution of the antenna to alter the spatial orientation or rotational behavior of the radiated electric field [67–69]. With the advancement of high-frequency communication systems, there is a growing demand for simultaneous frequency agility and polarization matching, giving rise to frequency and polarization-reconfigurable textile antenna technology. This technology holds significant value in improving spectral efficiency and enhancing anti-interference capabilities. Salleh et al. [84] developed a fully textile-based dual-reconfigurable (frequency and polarization) antenna. The design employs a truncated patch structure to achieve circular polarization, while frequency tuning is realized by adjusting the position of slots in the ground plane. Three PIN diodes are integrated into the ground plane slots, enabling dynamic control of the effective slot length through bias voltage. Additionally, 12 direct current (DC)-blocking capacitors are placed beneath the slots to ensure RF current flow while preventing DC interference. Experimental results demonstrate that the antenna can achieve flexible switching between 1.575 GHz (circular polarization) and 2.45 GHz (linear polarization).

**Fig. 4.**
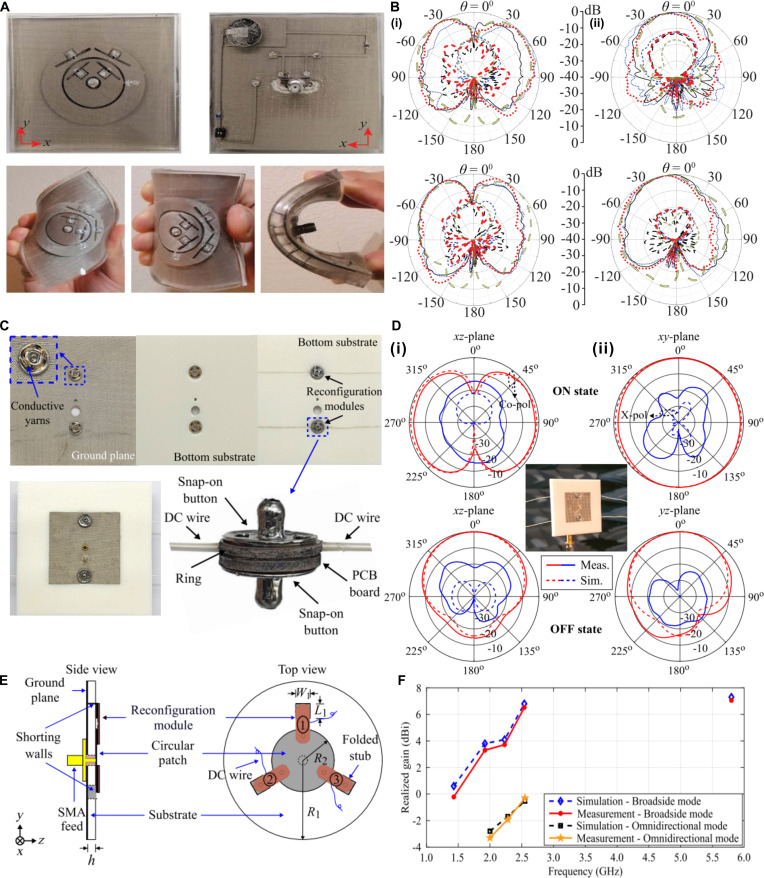
Textile radiation pattern-reconfigurable antennas. (A) Photograph of conductive textile-polymer composite antenna. (B) Radiation patterns of antenna (i) when all diodes are on at the *XZ*-plane and *YZ*-plane, and (ii) when all diodes are off at the *XZ*-plane and *YZ*-plane. Reproduced with permission from [79]. Copyright 2021, IEEE. (C) Photograph of the textile antenna integrating RF-switch ICs. (D) Radiation patterns of the antenna (i) in the on-state (ii) and in the off-state across the *XZ*-plane and *XY*-plane. Reproduced with permission from [80]. Copyright 2023, IEEE. (E) Schematic representation of the multifunctional reconfigurable textile antenna. (F) Realized gain of the frequency- and pattern-reconfigurable antenna under broadside and omnidirectional radiation conditions across multiple operating frequencies. Reproduced with permission from [83]. Copyright 2023, IEEE.

The radiation pattern, characterizing the spatial distribution of antenna radiation energy, critically determines key performance metrics including coverage range, gain, and interference immunity. Dynamic pattern reconfiguration has become increasingly important for wearable RF systems to achieve adaptive communication coverage and target tracking. Current pattern-reconfiguration approaches primarily involve multi-feed excitation with dynamic phase control [87,88], integration of RF switches to manipulate current distribution [70,71], or mechanical deformation of antenna structures. Pattern reconfiguration typically requires overlapping multiple resonant modes, which significantly increases structural complexity and presents nontrivial fabrication challenges for textile-based implementations. Recent developments of pattern-reconfigurable textile antennas [79–82] have demonstrated innovative solutions to achieve pattern reconfigurability while maintaining wearability. Yan and Vandenbosch [81] proposed a 2.45-GHz textile antenna using felt substrate and conductive fabric, incorporating 6 PIN diodes along the patch edges to control via states. When diodes are forward-biased, the vias establish low-impedance connections to ground, forming an inductor–capacitor (LC) resonant network with the patch’s inherent capacitance to excite zeroth-order resonance (ZOR) mode, producing monopole-like omnidirectional radiation. With diodes in the off-state, the antenna operates in the +1 resonance mode. This metamaterial-inspired design enables flexible pattern switching at a fixed frequency through dispersion engineering of the transmission line characteristics. Similarly, a complementary approach by Mohamadzade et al. [79] employed a PDMS-conductive fabric composite to fabricate a circular patch antenna (Fig. 4A). Four PIN diodes are integrated on the radiating patch with DC bias lines embedded in the ground plane. Selective activation of these diodes perturbs the balanced radial current distribution, enabling reconfiguration between the TM02-mode monopole-like pattern and broadside radiation (Fig. 4B). While these designs successfully demonstrate stable dual-mode operation in wearable environments, PIN diode-based implementations suffer from notable limitations, including significant insertion loss in the conducting state and limited power handling capacity. To address these issues, Fumeaux’s research group [80] developed a radiation pattern-reconfigurable textile antenna by integrating RF-switch integrated circuits (ICs) (Fig. 4C). The antenna was fabricated using a PF-4 foam substrate combined with conductive fabric, where 2 button-style switches containing embedded RF-switch ICs were mounted on the textile patch and connected to the ground plane through via holes. As demonstrated in Fig. 4D, in the switch-off state, the RF-switch functions as a parallel circuit between capacitors and resistors, effectively disconnecting the patch from the ground plane and resulting in a broadside radiation pattern similar to that of a conventional half-wave patch antenna. In the switch-on state, the RF switch exhibits extremely low resistance, transforming the radiation pattern into an omnidirectional mode characteristic of monopole antennas by applying a bias voltage. This innovative design effectively addresses the limitations of PIN diode-based approaches while maintaining the flexibility required for wearable applications.

The performance of wearable antennas is significantly compromised by mechanical deformations and environmental variations. To address these challenges while achieving universal communication capability for both on-body and off-body scenarios, as well as maintaining stable operation under deformation and positional changes, researchers have developed sophisticated multi-modal reconfigurable textile antennas. These advanced systems integrate multidimensional control of frequency, polarization, and radiation pattern, enabling adaptive multifunctional operation in complex EM environments. Fumeaux’s research group [83] used the processing method and near-field coupling feeding method of the aforementioned integrated conductive fabric with the reconfigurable modules on the PF-4 foam substrate, and achieved multidimensional control by regulating the varactor diodes of the 3 reconfigurable modules (Fig. 4E). Firstly, they demonstrated a frequency and textile pattern-reconfigurable antenna capable of switching between edge-radiating and dipole-like modes with impressive frequency-tuning ranges of 57.3% and 27.2% for each radiation mode, respectively (Fig. 4F). Secondly, they demonstrated a frequency and polarization-reconfigurable textile antenna offering a 31.9% frequency-tuning range while maintaining linear polarization reconfigurability at 0°, 120°, or 240° orientations. This work represents an advancement from their earlier single-function reconfigurable textile antennas, achieved through the strategic integration of additional reconfigurable modules to realize more complex, multi-parameter adaptive control in practical wearable applications.

### Textile rectifying antennas

Textile-based active antennas for energy conversion primarily focus on rectifying antennas (rectennas) [32,89–94]. With advancements in flexible wearable technologies, there is a growing demand for battery-free active devices to enable more comfortable wearing experiences and enhanced safety. In today’s IoT-rich spectrum environment, RF energy harvesting demonstrates broader applicability across all temporal and environmental conditions compared to traditional energy solutions. Consequently, textile-based RF systems utilizing RF energy harvesting have become a key research focus. The core component determining energy conversion efficiency is the rectenna, which performs the critical function of converting RF energy into DC power. A rectenna system consists of 2 essential parts: an energy-capturing antenna and a rectifier circuit with high-frequency filtering capabilities. The performance of rectennas depends not only on the radiation efficiency of the antenna but also on the impedance matching network. Here, we focus specifically on nonreconfigurable textile rectennas. The significantly lower power conversion efficiency (PCE) of fully textile-based rectennas compared to rigid counterparts stems from 3 fundamental limitations inherent to flexible substrates [31,32,90]. Firstly, the low dielectric constant of textile materials necessitates larger physical dimensions in impedance matching networks, inevitably increasing conductor losses. Secondly, the nonlinear characteristics of rectifying diodes exhibit greater performance degradation under mechanical deformation. Thirdly, impedance mismatch becomes more pronounced in deformable structures due to dimensional instability.

**Fig. 5.**
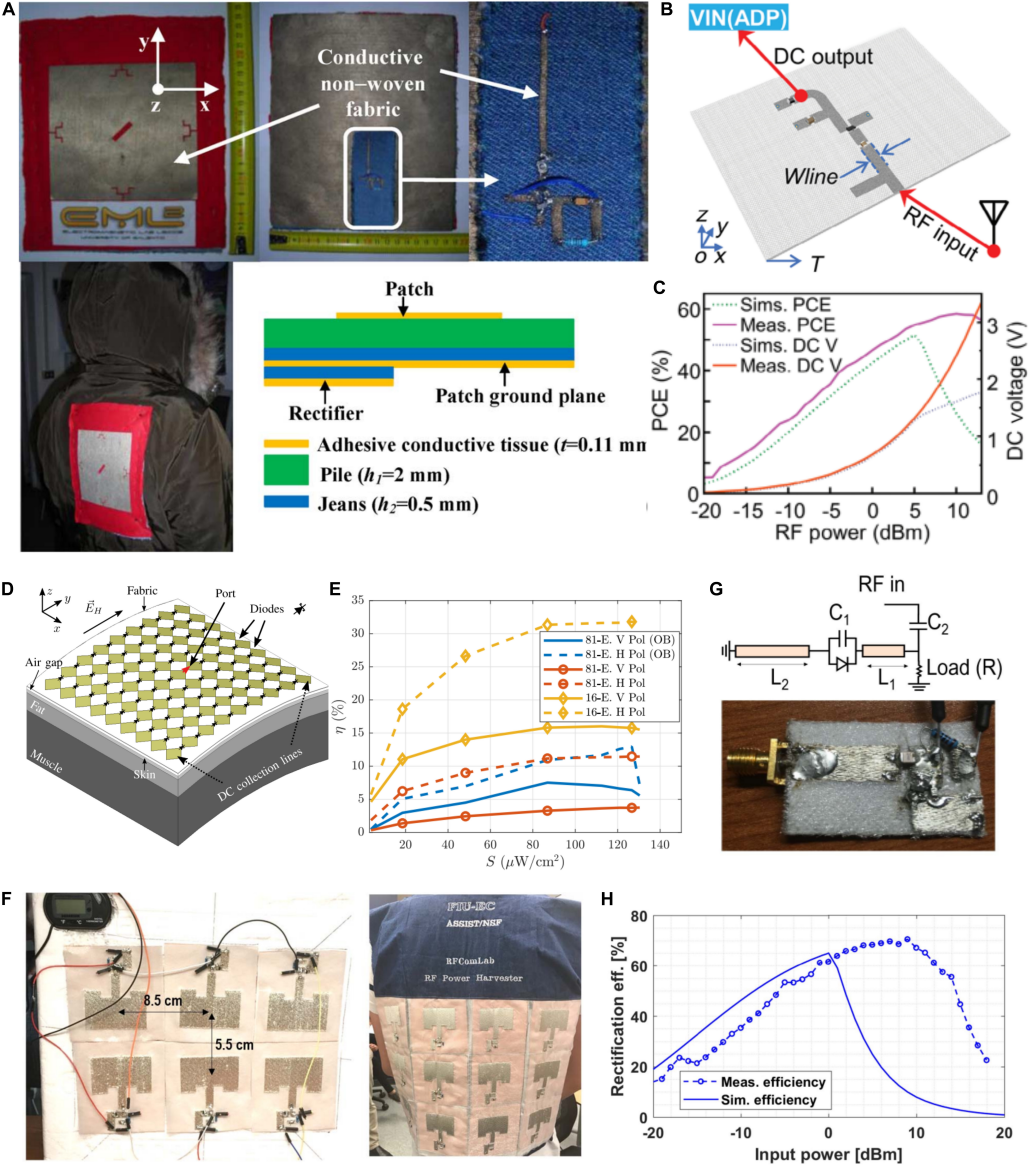
Textile-based rectifying antennas. (A) Photograph and structure composition of UHF textile rectennas. Reproduced with permission from [90]. Copyright 2013, IEEE. (B) Rectifying circuit of an all-fabric hybrid energy harvester. (C) DC output and PCE of the fabricated rectifying circuit across a range of input power levels. Reproduced with permission from [31]. Copyright 2024, John Wiley & Sons Inc. (D) Simulation geometry for RF-harvesting tightly coupled 9 × 9 rectenna array tee-shirt. (E) Measurement results of PCE versus incident power density at 2.9 GHz under a 2-kΩ DC load. Reproduced with permission from [89]. Copyright 2020, IEEE. (F) Photographs of the 6-element textile-based rectenna arrays used for the RF harvesting system. (G) Schematic and photograph of a textile-based rectifying circuit. (H) Measurement results for the RF-DC conversion efficiency up to 70%. Reproduced with permission from [32]. Copyright 2020, IEEE.

Early textile-based rectennas often relied on a hybrid architecture that integrated textile antennas with rigid PCB- or FPCB-based rectifiers [95–99], which significantly undermined the key wearable attributes of softness, lightweight, breathability, and flexibility. Monti et al. [90] proposed a fully textile rectenna operating in the 860- to 918-MHz UHF band (Fig. 5A). The device architecture employs a multilayer textile structure bonded by a thermo-adhesive layer, beginning with a rectangular patch antenna with a gain of 4.6 dB as the radiating element and a ground plane that serves as the intermediate layer. The antenna employs a compact design strategy by incorporating notches at all 4 corners of the rectangular patch, effectively reducing the overall size of the low-frequency antenna. The foundation of the system incorporates a full-wave bridge rectifier circuit through the matching of lumped components at the final layer, whose maximum PCE is 50% with an incident power density of 14 μW/cm^2^ at the frequency of 876 MHz. Researchers further optimized textile rectennas by designing antennas, rectifiers, and matching circuits, improving flexible material performance. Wagih et al. [91] proposed a textile rectenna with a maximum PCE of 50% in an energy harvesting system operating at a power density of less than 1 μW/cm^2^. This breakthrough was realized through laser-cut conductive fabric forming a dual-polarized, low-profile patch antenna integrated with FPCB-based multi-frequency rectifiers on a lossy felt substrate. While these designs demonstrate promising energy harvesting capability, the partial reliance on nontextile rectifier components indicates that achieving a fully textile-integrated rectenna remains an important research direction. To solve this problem, Lu’s research group [31] developed a 2.4-GHz circular patch rectenna through precision laser-cut copper fabric transferred onto textile substrates. Figure 5B and C depicts that the rectifying circuit utilizes a first-order Greinacher-type full-wave rectification topology, directly integrating with antenna by stub matching method, demonstrating outstanding performance with an antenna gain of 4.4 dBi and a remarkable PCE reaching 58% at an RF input power of 10 dBm.

Furthermore, to enhance energy harvesting efficiency by expanding the effective reception area of antenna in low-power density environments while simultaneously optimizing impedance matching and frequency response, research on a textile rectenna array with higher gain than a single antenna element has been increasingly prominent in the RF energy harvesting field. As shown in Fig. 5D, Estrada et al. [89] fabricated 4 × 4 and 9 × 9 bowtie-shaped rectenna arrays by screen-printing silver paste on cotton fabric. By integrating rectifying diodes at ^1^/_6_-wavelength intervals in the gaps between array elements, where the diodes were connected in series along the co-polarized electric field vector to the antenna feed points with each row in parallel to the DC load, the arrays achieved RF energy harvesting with a power density of 4 to 130 μW/cm^2^ across a frequency range of 2 to 5 GHz (Fig. 5E). Testing showed that the impedance matching and radiation gain of the textile-based device remained unaffected even when placed 1 mm away from the skin. Additionally, the design can be expanded to support dual-polarization operation through the strategic placement of additional diodes at selected feed points. Although this study systematically evaluates the performance of integrated textile rectenna arrays in wearable cloths, advancing their practicality for on-body energy harvesting applications, the way of directly connecting the rectifying diode to the antenna element and relying solely on mutual coupling effects between array elements to adjust the input impedance exhibits limitations, as the RF-DC PCE is typically lower than designs incorporating dedicated impedance matching networks. Therefore, Vital et al. [32] demonstrated rectenna arrays that incorporate impedance matching circuits and rectifying circuits. From Fig. 5F, the silver-coated copper strands were embroidered onto the textile substrate according to the designed antenna pattern and circuit structure, with electronic components securely fixed. Each rectenna consists of a single 2.45-GHz patch antenna connected to a T-type matching network and then to a rectifying circuit. As shown in Fig. 5G, the individual rectenna achieved a gain of 6.5 dBi and 70% PCE. To practically test the energy harvesting capability, the fabricated 2 × 3 rectenna array could still harvest 80 μW of DC power when placed 60 cm away from a 500-mW RF source (Fig. 5H). This work not only provides a large-scale fabrication method for subsequent all-textile active RF devices but also demonstrates the rectenna’s capability for long-distance, highly efficient energy harvesting.

Table 1 summarizes representative textile-based RF energy harvesting devices in terms of operating frequency, integration architecture, impedance matching network, rectifier topology, and energy conversion performance. Most reported textile rectennas operate in the sub-GHz industrial, scientific, and medical (ISM) band (0.86 to 0.92 GHz) and the 2.45-GHz ISM band, benefiting from a favorable trade-off between propagation loss, antenna size, and suitability for wearable applications. Recent studies have further extended textile platforms to multiband and wideband operation, reflecting growing interest in broadband ambient RF energy harvesting. Laser-cut copper fabric antennas are most widely adopted due to their low ohmic loss and fabrication repeatability, whereas embroidered conductive thread antennas provide superior mechanical compliance at the expense of reduced RF efficiency. The choice of rectifier topology is strongly dependent on the input power level and system requirements. Voltage doubler rectifiers are typically employed under ultra-low input power conditions to achieve higher DC output voltage, while single-diode half-wave rectifiers offer the highest RF-to-DC conversion efficiency at moderate power levels owing to their low conduction loss and simple structure. Full-wave bridge rectifiers are more suitable for higher input power scenarios, particularly when polarization or phase insensitivity and low output voltage ripple are required, albeit with increased diode loss. For multiband energy harvesting, parallel rectifying branches optimized for different frequency bands are commonly implemented to enable efficient multi-frequency rectification. It should be noted that energy harvesting efficiency is characterized using different metrics across the literature. Textile metasurfaces and antenna arrays often report overall system harvesting efficiency, whereas rectennas typically evaluate RF-to-DC PCE of the rectifier. Therefore, careful distinction is required when comparing reported efficiency values.

**Table 1.** Comparative performance analysis of textile energy harvesters

Type	Frequency (GHz)	Integration architecture	Impedance matching network	Rectifier topology	Overall efficiency	Antenna maximum gain (dBi)	RF-to-DC PCE	Diode	Ref.
Textile rectenna	0.860–0.918	Laser-cut copper fabric rectenna	Distributed-element matching (single microstrip line matching)	Full-wave bridge rectifier	N/A	4.6	50%	HSMS-285X	[90]
	2.45	Fabric antenna with rigid rectifier (duroid-5880)	Distributed element matching (L-type)	Single-diode rectifier	N/A	8.1	33.6%	SMS7630	[98]
	0.7–2.7	Fabric antenna with FPCB-based rectifier	Lumped-element matching (L-type)	Single-diode rectifier	N/A	6.5	41.8%	SMS7630	[91]
	2.45	Laser-cut copper fabric rectenna	Distributed-element matching (T-type)	Voltage doubler rectifier	N/A	4.4	58%	SMS7630	[31]
	2.45	Embroidered conductive thread rectenna array	Lumped and distributed element matching (L-type)	Single-diode rectifier	N/A	5	70%	SM57630	[32]
	2–5	Laser-cut copper fabric rectenna	Distributed-element matching	Single-diode rectifier	32%	N/A	N/A	SMS7630	[89]
Textile metasurface	2.9, 4.72, 5.37	Laser-cut copper fabric rectenna	Lumped-element matching (L-type)	Single-diode rectifier	43%	N/A	N/A	SMS7630	[25]

## Textile Active Metasurface

While textile-based passive metasurfaces [100–103] enabled static wavefront shaping, their EM functionalities are irrevocably fixed upon fabrication, with research challenges mainly associated with manufacturing precision and environmental robustness. To overcome this intrinsic static limitation, subsequent efforts have explored textile-based active antennas, in which discrete active components are integrated to enable dynamic control of EM transmission and reception. However, such antenna-centric approaches primarily manipulate the EM response at the device or port level, offering limited capability for spatially distributed or regional wavefront control across a continuous aperture. To transcend these constraints and realize truly intelligent and adaptive textile-based RF platforms, research attention has increasingly shifted toward textile-based active metasurfaces [10,25,104]. By introducing sub-wavelength, locally reconfigurable unit cells, this paradigm enables distributed and fine-grained control of EM wavefronts, addressing the fundamental challenges of large-area system integration and dynamic EM manipulation on flexible textile substrates.

**Fig. 6.**
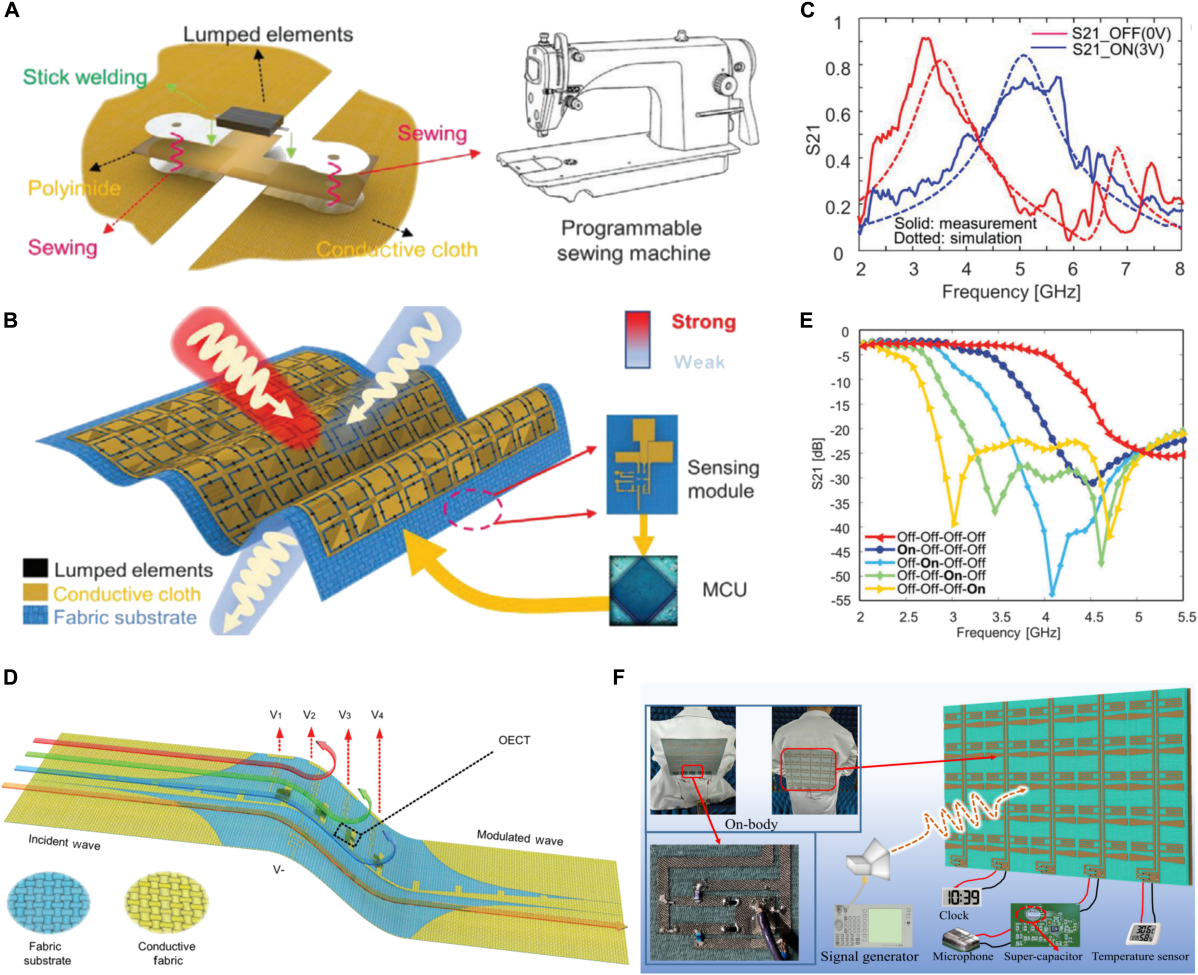
Textile-based metasurfaces. (A) Welding-sewing method: Schematic of the fabrication process, involving cutting-transfer patterning of conductive cloth, polyimide film-based lumped element welding, and sewing-based layer integration. Schematic of the welding-sewing method. (B) FSM integrating a sensor module, MCU, and tunable metasurface. (C) EM response of fabric-based tunable metasurface. Values of 0 and 3 V are used to control the “off” and “on” states of the metasurface according to the intensity of external illumination. Reproduced with permission from [10]. Copyright 2023, John Wiley & Sons Inc. (D) Schematic of the TFMF based on OECTs. (E) S21 parameters of the TFMF waveguide at the different states of the OECTs. Reproduced with permission from [105]. Copyright 2023, John Wiley & Sons Inc. (F) Schematic of the EMEH platform to power the microphone and temperature sensor. Reproduced with permission from [25]. Copyright 2025, IEEE.

Hossain et al. [104] presented a reconfigurable textile metamaterial unit featuring a decagonal split-ring resonator and a slotted ground plane integrated with RF varactor diodes. The conductive pattern of the array unit was formed by laser-cutting textile and thermally transferring it onto a felt substrate, while lumped components were connected to both sides of the ground plane on the back using conductive epoxy. Experimental results demonstrate that the transmission coefficient, equivalent permittivity, equivalent permeability, and refractive index of the metasurface can be dynamically tuned across different frequency bands by varying the bias voltage applied to the varactor diode. However, integrating more complex functionalities onto textiles poses distinct challenges, including the intolerance of fabrics to high-temperature soldering and difficulties in reliably attaching electronic components. Therefore, Lu’s group [10] innovatively proposed a “welding–sewing” strategy to prepare the fabric-based smart metasurface (FSM). As shown in Fig. 6A and B, this method involved first soldering PIN diodes onto a polyimide interposer, which was then securely connected to copper fabric electrodes through precision stitching, thereby avoiding thermal damage to the fabric. FSM included the antenna to receive the EM waves, the sensing module to recognize the incident power intensity, and an MCU to control the PIN diodes to change the operation mode of FSM. In Fig. 6C, transmission parameters (S_21_) have demonstrated that, under low-power conditions, incoming waves transmit through the FSM for normal communication with backend equipment, whereas high-power waves are automatically reflected to provide adaptive EM protection. The FSM exemplifies a leap from passive adaptation to active decision-making in flexible EM protection and adaptive camouflage.

In pursuit of more fully flexible integration processes for RF textile devices, the aforementioned team [105] utilized OECTs to replace rigid lumped components, directly printing them onto fabric patterns to fabricate a tunable fabric microwave filter (TFMF) (Fig. 6D). Utilizing a laser-cutting and thermal transfer process, a spoof surface plasmon polariton (SSPP) structure was fabricated on polyester fabric, with printed OECTs serving as dynamic tuning units. In Fig. 6E, by modulating the gate voltage of the OECT, the device achieved multi-state electrically tunable S_21_ parameters within the frequency range of 2.7 to 5.5 GHz. The proposed TFMF not only demonstrates high flexibility in tuning performance but also exhibits excellent mechanical flexibility and conformability to the human body, offering a promising solution for frequency manipulation in next-generation wearable communication systems.

Beyond multifunctional EM wave manipulation of FSM or TFMF, Lu’s team [25] introduced an EM energy harvesting (EMEH) platform based on a fabric metasurface (Fig. 6F). Similar to the textile rectenna arrays introduced in Textile rectifying antennas, energy harvesting textile metasurfaces are also designed to convert ambient RF waves into usable DC power. The latter can be regarded as an evolution of the former, offering significantly higher integration and more advanced wave-manipulation capabilities. Their design employs a multi-band absorbing metasurface that combines bow-tie and zigzag dipole structures to capture ambient RF energy, which is then channeled through an integrated network to a rectifying circuit. Using laser-cut conductive fabrics for precise patterning and PDMS to encapsulate, the multi-band design of metasurface combines a bow-tie dipole (2.77 GHz), a zigzag dipole (4.53 GHz), and a second-harmonic resonance from the bow-tie interaction (5.27 GHz), allowing effective harvesting of ambient RF energy from diverse sources. They applied the platform to power temperature, humidity, or sound sensors, validating its practicality as an autonomous energy solution for self-sustained wearable electronics. These works demonstrate the functional enrichment of active fabric metasurfaces, which exhibit capabilities such as dynamically tunable frequency domains, power-aware adaptive EM protection, and ambient energy harvesting. Through innovatively adopted strategies including “cutting-transfer”, “welding-sewing”, and “Pin-extension”, these studies have successfully addressed the compatibility challenges between small-sized rigid components and commonly used flexible textile substrates. These advances propel textile-based metasurfaces from conceptual validation toward practical application in next-generation wearable textile electronics.

Despite significant progress, textile-based active metasurfaces remain at an early stage of system-level maturity. A primary challenge lies in ensuring electromechanical reliability under repeated deformation and real wearable conditions, where EM performance must be designed with textile mechanics to achieve long-term stability. In addition, current implementations mostly rely on predefined control schemes, limiting their adaptability. Future systems should incorporate closed-loop sensing and adaptive control to enable context-aware EM responses. Power autonomy is another key constraint, as the harvested energy from fabric platforms is often marginal for sustained operation, highlighting the need for co-designed metasurfaces that simultaneously support wave manipulation and energy harvesting. Finally, the absence of standardized evaluation metrics hampers cross-comparison and practical translation. Establishing unified criteria that jointly consider EM performance, mechanical durability, and system-level efficiency will be essential for advancing textile metasurfaces toward real-world wearable applications.

## Textile-Based Active RF System

With the rapid advancement of RF electronics and textile-related processing technologies, textile-based RF systems have emerged as a significant research focus in the fields of wearable health monitoring, energy harvesting, and human–computer interaction. Early research efforts were primarily dedicated to integrating conventional rigid electronic components with textiles in a flexible manner, employing techniques combining fabric-embroidered electrodes or fabric-coupled coils with PCB or FPCB circuit boards on the fabric substrate to achieve preliminary system integration and wireless communication functions. Monti et al. [90] developed a garment-embedded patient monitoring system for detecting sudden infant death syndrome. Both knitted and woven stainless steel electrodes, and the 132-kHz inductively coupled coil for wireless power transfer and bidirectional data communication are all fabricated via embroidery technology. Wireless transceiver and battery were integrated by fabric circuit board (FCB) technology using knitted conductive wire to connect the electrodes and the coil. Serving as an early pioneering demonstration of a wireless textile system, this work has laid important technical foundations for the later development of integrated and self-powered medical wearable textile devices.

**Fig. 7.**
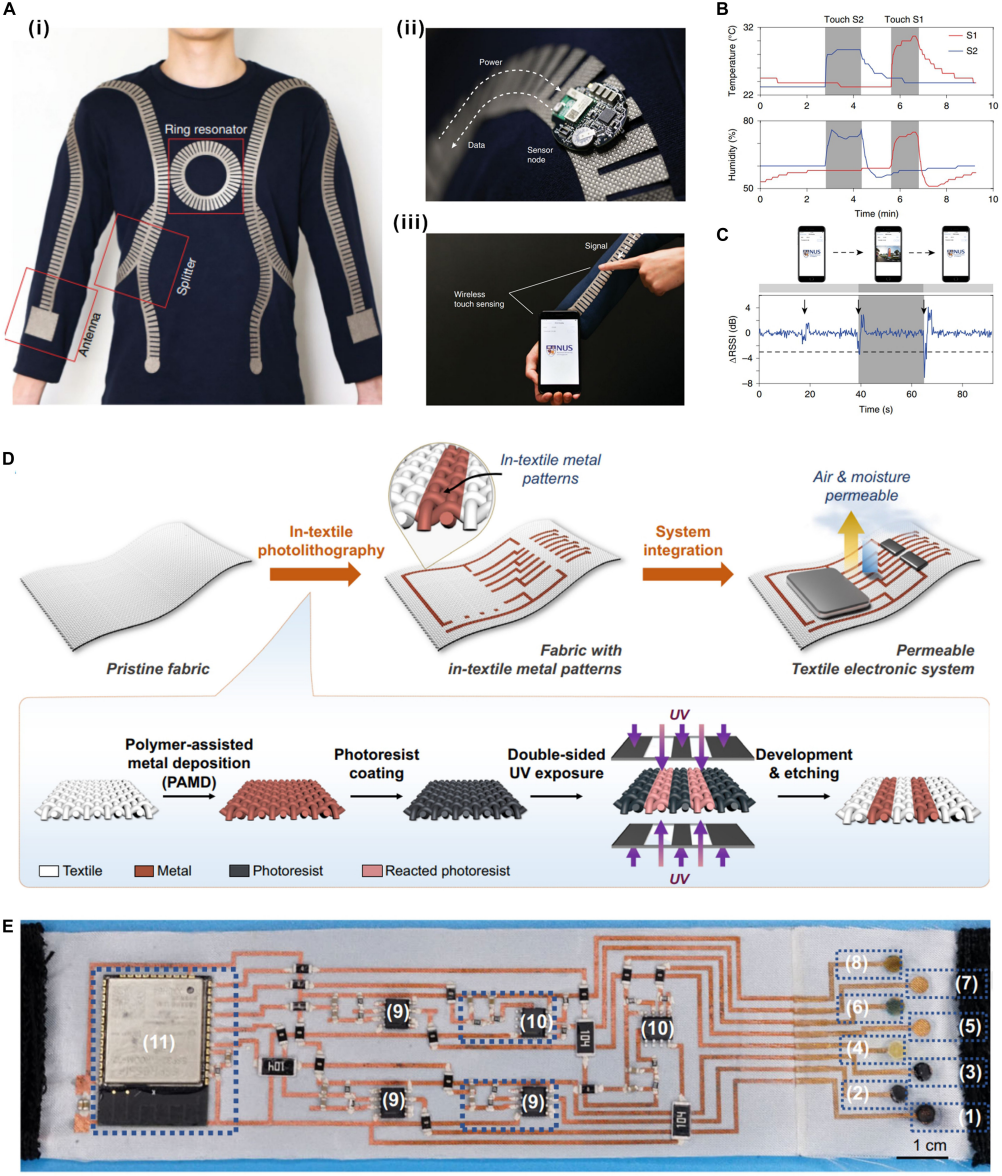
Textile-based active RF system, part 1. (A) (i) Metamaterial textile network integrating splitters, antennas, and a ring resonator. (ii) SSPP metamaterial textiles power the Bluetooth sensing module for PCB fabrication. (iii) Sensing data via Bluetooth for real-time display on a smart terminal. (B) Shoulder (S_1_) and wrist (S_2_) temperature data detected by the system are displayed on the smartphone. (C) Changes in received signal strength indicator signals with the finger approaching change the display of the image. Reproduced with permission from [30]. Copyright 2019, Springer Nature. (D) Entire preparation process of in-textile photolithography technology and system integration. (E) Photograph of an integrated multiplexed biosensing headband for wireless sweat sensing. Reproduced with permission from [17]. Copyright 2024, Springer Nature.

Driven by continuous advances in textile processing technologies, textile health monitoring systems based on wireless power transfer are progressively evolving toward greater integration, improved interconnect stability, and stronger systemic coherence. Ho’s research group [15,16,30] has conducted a series of innovative studies in the field of textile-based wireless systems for human health monitoring. Their early work focused on the development of metamaterial textiles based on SSPP structures and near-field communication (NFC) antennas for wireless power transfer [30]. The SSPP structure is an artificially designed periodic groove or hole array that effectively mimics the surface plasmon polariton effect of metals at optical frequencies while extending its operational range to lower frequencies such as microwaves and terahertz waves. A key advantage of this structure is its ability to efficiently support tightly bound RF surface wave propagation along the material surface, with EM energy highly localized near the structure, thereby significantly reducing radiative losses into free space. Based on this principle, the metamaterial textile serves as a high-performance transmission line, enabling efficient near-field wireless energy transfer and sensing. As illustrated in Fig. 7A to C, EM energy can be guided and propagated through the SSPP transmission line integrated into a long-sleeved sweater toward the wrist. FPC circuits integrating the rectenna and pressure sensor are placed on the wrist and chest area. After wirelessly receiving RF energy, the FPC circuit can display the flashing of light-emitting diode (LED) based on pulse or heart rhythm signals detected by the pressure sensor, achieving physiological signals for real-time visualization and monitoring of physiological signals. This work also showed that the distributed wireless sensor network for human–computer interaction has been demonstrated. By bringing a rigid PCB module integrated with temperature, humidity sensors, and Bluetooth communication close to the SSPP metamaterial textiles, sensing data can be displayed in real time on a smart terminal. Because of the strong EM confinement capability of the SSPP structure, the wireless transmission efficiency of this system is improved by more than 3 orders of magnitude (>30 dB) compared to traditional radiative communication while confining signals within 10 cm of the body surface.

Unlike the approach of directly integrating PCB or FPCB circuit boards with textile-based RF devices, many research teams have focused on creating circuits directly on fabrics. However, the direct fabrication of high-precision, fully textile-based circuits on rough and porous fabric substrates remains a significant technological challenge. To address this core technological problem, Zheng’s research group [17] recently reported a revolutionary in-textile photolithography technology, combining polymer-assisted metal deposition and double-sided ultraviolet lithography (Fig. 7D), which enables the metal to be coated on the surface of each fiber inside the fabric with an accuracy of <100 μm. Thus, conductive patterns can be completely penetrated into the 3-dimensional (3D) porous framework of the fabric. The ventilation rate of the textile circuit is 21 times higher than that of traditional screen-printed circuits. Further, a fully integrated multi-channel sweat-sensing headband was fabricated to verify this processing technology (Fig. 7E). Multiple potential-type sensors for detecting pH, Na^+^, and K^+^, as well as amperometric enzyme sensors for detecting glucose and lactic acid, were all integrated on the fabric substrate flexibly. Although its wireless communication circuitry relies on rigid RF modules, it provides a crucial manufacturing platform for seamless and breathable high-precision integration of complex silicon-based chips, lumped elements, and sensing electrodes into smart textiles.

**Fig. 8.**
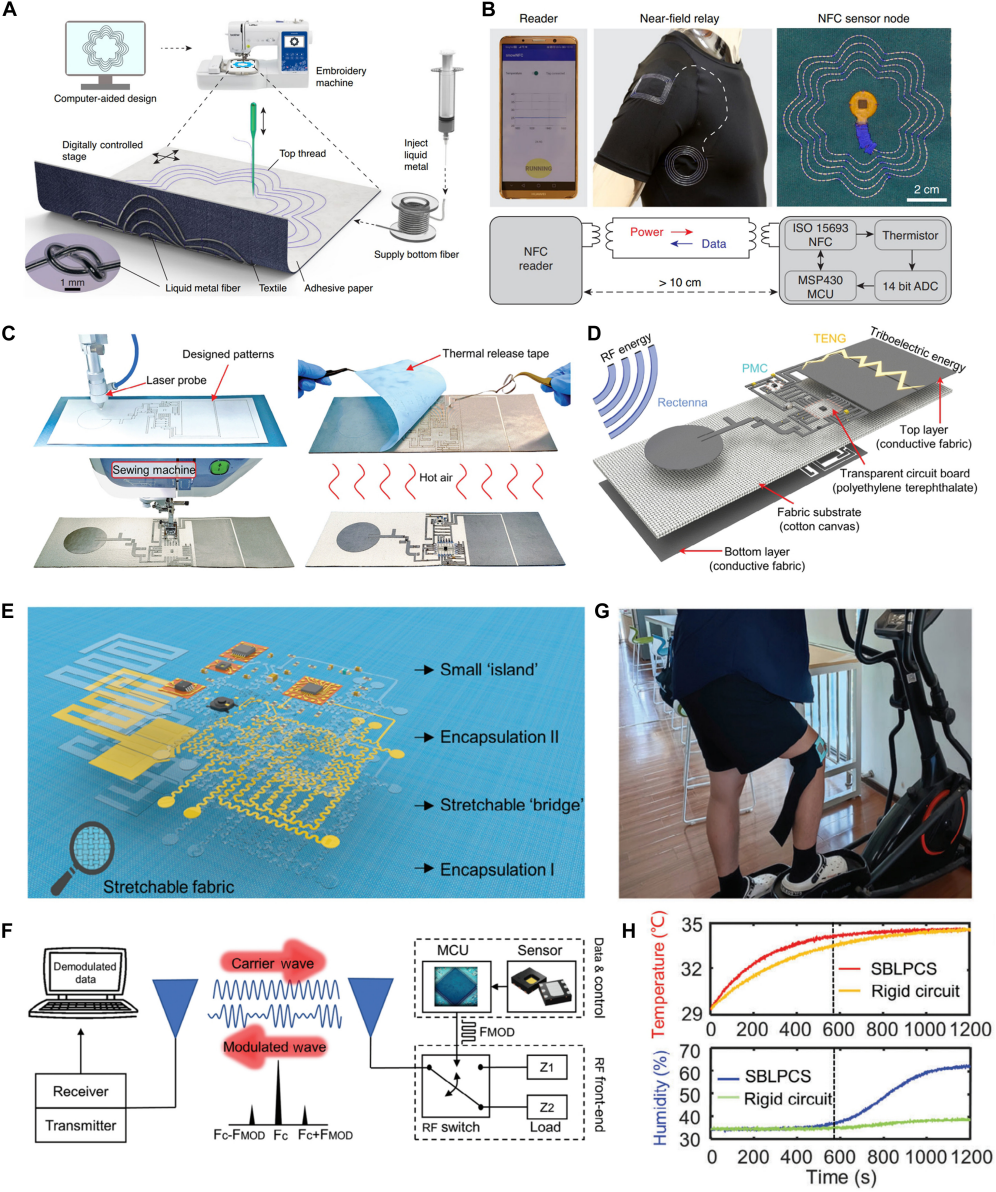
Textile-based active RF system, part 2. (A) Demonstration of the digital embroidery process. Computer-controlled embroidery of liquid metal fibers transfers the pattern of the NFC antenna onto the textile substrate. (B) Top: Photograph of textile thermal monitoring system, which wirelessly transmits data from a digitally embroidered thermal sensor placed under the arm to a custom application on a smartphone worn on the arm via NFC. Bottom: Architecture of the textile thermal monitoring system. Reproduced with permission from [16]. Copyright 2022, Springer Nature. (C) Process of FCB-SMT technology, which includes cutting, transfer, and thermal of metallic patterns, stitching of metallized vias, and assembly of lumped components. (D) Schematic of a wearable all-fabric hybrid energy harvester to simultaneously harvest radiofrequency and triboelectric energy. Reproduced with permission from [31]. Copyright 2024, John Wiley & Sons Inc. (E) Schematic of the fabric-based stretchable and breathable backscattered monitoring system (SBBS). (F) Illustration of a backscatter communication module: A transmitter emits a single-frequency carrier wave, which is modulated by the backscatter device via an RF switch connected to an antenna. The resulting reflected signal is subsequently decoded by a receiver to retrieve the data. (G) SBBS worn around the knee of the human body to monitor temperature and humidity. (H) Temperature and humidity curves recorded during a 2-min interval with the SBBS worn around the knee. Reproduced with permission from [18]. Copyright 2024, John Wiley & Sons Inc.

Furthermore, Ho’s group [16] focused on using liquid metal fibers to achieve deeper and more monolithic integration of electronic functions into textiles for near-field wireless power and communication. They proposed a digital embroidery strategy based on liquid metal, in which a gallium–indium–tin alloy is encapsulated within perfluoroalkoxyalkane polymer tubing to form fiber-like conductors that combine high electrical conductivity with mechanical flexibility. As demonstrated in Fig. 8A, using computer numerically controlled embroidery, pre-optimized circuit patterns were accurately integrated into textile substrates. In their study, the authors successfully developed a wirelessly powered smart shirt capable of continuous underarm temperature monitoring. A key breakthrough lies in the use of embroidered liquid metal coils as NFC antennas operating at 13.56 MHz to wirelessly receive the data from a digitally embroidered thermal sensor in the underarm, achieving unified integration of sensing, power supply, and communication functions (Fig. 8B). This work lays a solid foundation for next-generation fully flexible, seamlessly integrated, and clinically viable wearable textile-based RF electronics.

Lu’s group [31] developed a fabric circuit board quasi-surface mount technology (FCB-SMT). This approach was used to fabricate a fully textile-based, wearable hybrid energy harvester prototype capable of simultaneously harvesting both RF and triboelectric energy. As illustrated in Fig. 8C, the FCB-SMT process involves cutting patterned conductive fabric, transferring and thermally releasing it onto a textile substrate, stitching metallized vias using a sewing machine, and finally soldering lumped components onto the fabric-based circuitry. Within this system, a 2.4-GHz all-fabric rectenna with a gain of 4.4 dBi and a PCE of 58% harvests ambient RF energy, while a fabric-based thermoelectric generator effectively captures kinetic energy. To effectively resolve the critical challenge of mismatched output characteristics between different energy sources in hybrid energy harvesting systems, a specially designed fabric-based power management circuit was designed by incorporating maximum power point tracking, charge protection, and undervoltage lockout functionality (Fig. 8D). This work offers a practical and scalable solution for low-cost, high-volume industrial production from an individual antenna to a complete textile-based RF circuit.

Traditional RF systems, mostly utilizing active communication mechanisms such as Bluetooth or LoRa that wirelessly transmit data, result in high power consumption to recharge or replace batteries frequently in wearable devices. Besides, the relatively large and rigid communication module also poses obstacles to the comfort and safety of wearing, as shown in Fig. 7E. To address the critical challenges of limited stretchability and high-power consumption, Lu’s research group [18] integrated a low-power backscatter communication mechanism with a textile-based RF system, developing a fully stretchable, breathable, and low-power physiological monitoring system (SBBMS) with a total power consumption of only 6.1 mW (Fig. 8E to H). By employing a printing-cutting-transfer technology combined with an "island-bridge" strategy, high-precision serpentine copper circuits on fabric substrates can be achieved, overcoming common limitations such as poor interlayer connectivity and insufficient breathability found in conventional textile-based electronics.. Furthermore, the incorporation of a zinc-ion hydrogel battery and a multi-source energy management architecture ensures a safe, flexible, and sustainable power supply, also supporting energy harvesting to extend operational duration.

**Fig. 9.**
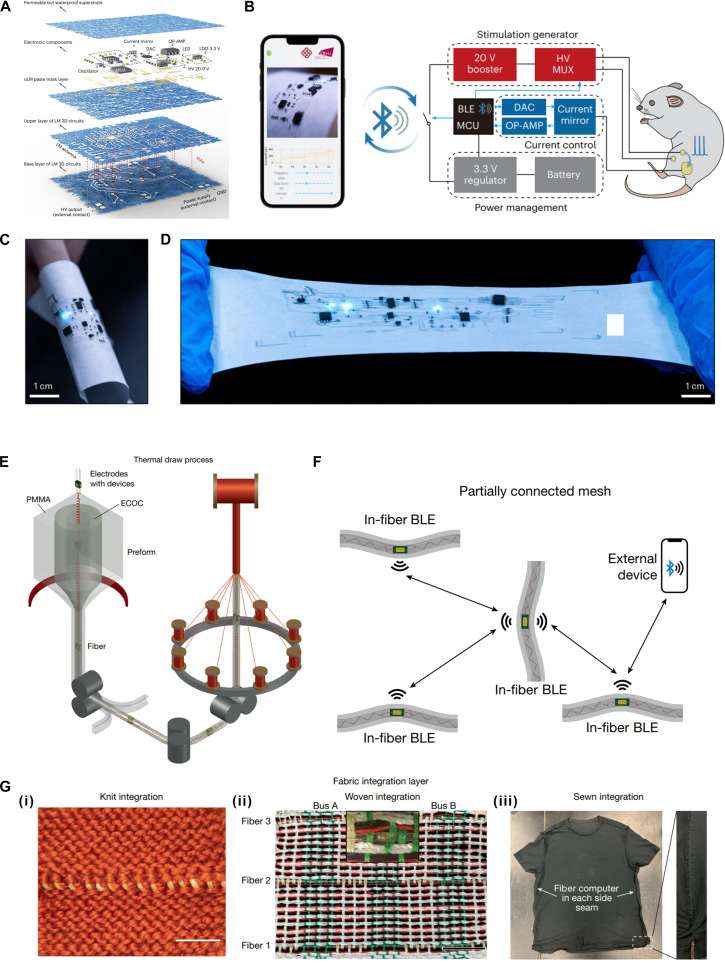
Textile-based active RF system, part 3. (A) Exploded schematic of P3D-eskin. (B) Schematic of the P3D-eskin. Electrical stimulation is applied to the biceps femoris muscle of a rat, and the corresponding electromyography signals are recorded by a smartphone via an liquid metal microelectrode. (C) Photograph of P3D-eskin at the bent state. (D) Photograph of P3D-eskin at the stretch rate of 550%. Reproduced with permission from [14]. Copyright 2024, Springer Nature. (E) Schematic of thermal draw fiber computer fabrication process followed by braiding. (F) Schematic of a multi-fiber Bluetooth communication system employing a partially interconnected mesh network topology. (G) (i) Integrated fiber computer unit embedded in a knitted textile, incorporating an MCU, LED, and voltage regulator. (ii) Woven textile sample featuring 3 fiber computers aligned in the weft direction, interconnected by 2 waveguide buses running along the warp direction. (iii) Custom-modified garment with 2 fiber computers seamlessly incorporated into the side seams. Reproduced with permission from [9]. Copyright 2025, Springer Nature.

Although the SBBMS achieved certain stretchability through the combination of serpentine copper foil conductive traces and a stretchable fabric substrate, the inherent nonelastic property of the copper foil material restricts the overall stretchability of the system. The development of stretchable textile-based RF systems is moving toward greater stretchability in order to adapt to a wider range of scenarios. The recently reported all-fabric 3D integration strategy by Zheng’s research group [14] marks a significant advance in textile-based electronics. Their permeable, 3-dimensional electronic skin (P3D-eskin) incorporates photolithography, pattern transfer, and stencil printing to create high-resolution liquid metal circuits on fibrous substrates (Fig. 9A). A hybrid liquid metal solder ensures robust connections between rigid components and stretchable interconnects within a 3D architecture, while electrospun fiber mats provide permeable encapsulation. As illustrated in Fig. 9C and D, the system exhibits exceptional stretchability up to 1,500%, reduced 54% thickness, and enhanced 60% softness compared to conventional PDMS-based e-skins. It demonstrates practical functionality in wireless transcutaneous stimulation and multi-point temperature sensing, supported by both 13.56-MHz NFC antenna and 2.4-GHz Bluetooth antenna, achieving long-range communication up to 15 m (Fig. 9B). This work provides valuable insights for the development of next-generation stretchable textile-based systems.

In 2025, Fink’s research group [9] has pioneered the integration of commercial microchips with helical copper microwires into a single elastic fiber using a foldable interposer and thermal drawing process, resulting in the first “fiber computer” capable of sensing, data storage, processing, and communication. In Fig. 9E, by interconnecting 8 microdevices via helical copper microwires, the team produced a machine-washable and highly stretchable fiber that exhibits over 60% elongation. As shown in Fig. 9F and G, incorporated with a 32-bit floating-point microcontroller, this programmable fiber independently performs edge computing tasks even when braided, woven, knitted, or sewn into garments. Through collaborative decision-making within an RF network, the system demonstrates enhanced distributed intelligence in applications such as human activity classification, where inference accuracy improved significantly from 67% to 95%. This work marks a significant advancement in the structural design of fiber-based electronic systems by integrating microchips, power management units, and communication modules within a single elastic fiber through a thermal drawing process. This innovation enables on-fiber computation and data storage while maintaining flexibility and washability, offering practical potential for smart garments, continuous health monitoring, and distributed textile-based IoT systems.

In summary, textile-based RF systems have evolved from initial partial integration of functional units into highly systemic, multidimensionally perceptive, and distributively intelligent all-textile electronic platforms (Table 2). From the Ho research group’s liquid metal digital embroidery [16], to the Zheng team’s high-precision in-textile photolithography [17], and further to the Lu group’s FCB-SMT process and low-power backscatter communication systems [18,31], numerous innovative approaches have continuously enhanced system integration, mechanical stretchability, and wearing comfort while significantly reducing power consumption and expanding functional capabilities. The P3D-eskin fabricated by Zhuang et al. [14] and the world’s first fiber computer developed by the Fink group [9] further demonstrate that smart textiles are transitioning from electronics on fabrics to a new paradigm of electronics as fabric.

**Table 2.** Summary of manufacturing techniques of textile-based RF systems

Type	Frequency	Power consumption	Manufacturing technique	Integration level	Best for	Cost	Ref.
RF sensing system	2.4 GHz, 13.56 MHz	20 V/2 mA	Screen printing	Impregnated into the textile	Large area, mass production, specified textile	Cheap	[14]
Antenna	2.4 GHz	N/A					[79]
Antenna	2.4 GHz	N/A					[105]
Filter	2.6–4.6 GHz	N/A					[34]
RF energy harvesting system	2.9, 4.72, 5.37 GHz	N/A	Laser cutting	Laminated onto surface of the textile	Large area, mass production, nonspecified textile	Cheapest	[25]
RF sensing system	915 MHz	6.1 mW					[18]
RF sensing system	2.4 GHz	100 mW					[30]
RF energy harvesting system	2.4 GHz	N/A					[32]
RF energy harvesting system	2.4 GHz	N/A					[31]
RF energy harvesting system	13.56 MHz	200 mW	Embroidery	Sewn into interior of the textile	Low-complexity, quasi-1D pattern on textile	Moderate	[15]
RF sensing system	13.56 MHz	N/A					[16]
RF sensing system	2.4 GHz	N/A	Photolithography	Fabric metallization	High-complexity, small-area pattern	Expensive	[17]
RF sensing system	N/A	N/A					[106]
RF sensing system	N/A	N/A	Thermal draw fiber	“Fiberization” to “sewing” approach	High integration, miniaturization, mass production	Most expensive	[9]

However, we emphasize that these advantages come with new and nontrivial challenges that are fundamentally different from those encountered in conventional wearable electronics. At the materials and processing level, stress-induced ohmic losses, interfacial instability between soft textile substrates and rigid electronic components, and long-term mechanical fatigue become increasingly pronounced as system size increases. At the device and circuit level, ensuring deformation-insensitive RF performance, particularly for antennas, matching networks, emerges as a critical requirement for maintaining system-level reliability under realistic wearing conditions.

In our view, the next stage of textile-based RF systems will be defined not by further increases in functional density but by the co-optimization of EM performance, mechanical compliance, and energy sustainability at the system scale. Although challenges remain in achieving high-precision RF fabrication, robust interconnections, and stable energy management on complex textile substrates, the parallel development of multiple disruptive technological pathways, together with continued innovation in materials, processing strategies, and system architectures, is expected to drive textile-based RF systems toward fully self-powered, seamlessly integrated, edge-intelligent, and long-range communication-enabled textile platforms.

## Conclusion and Outlook

**Fig. 10.**
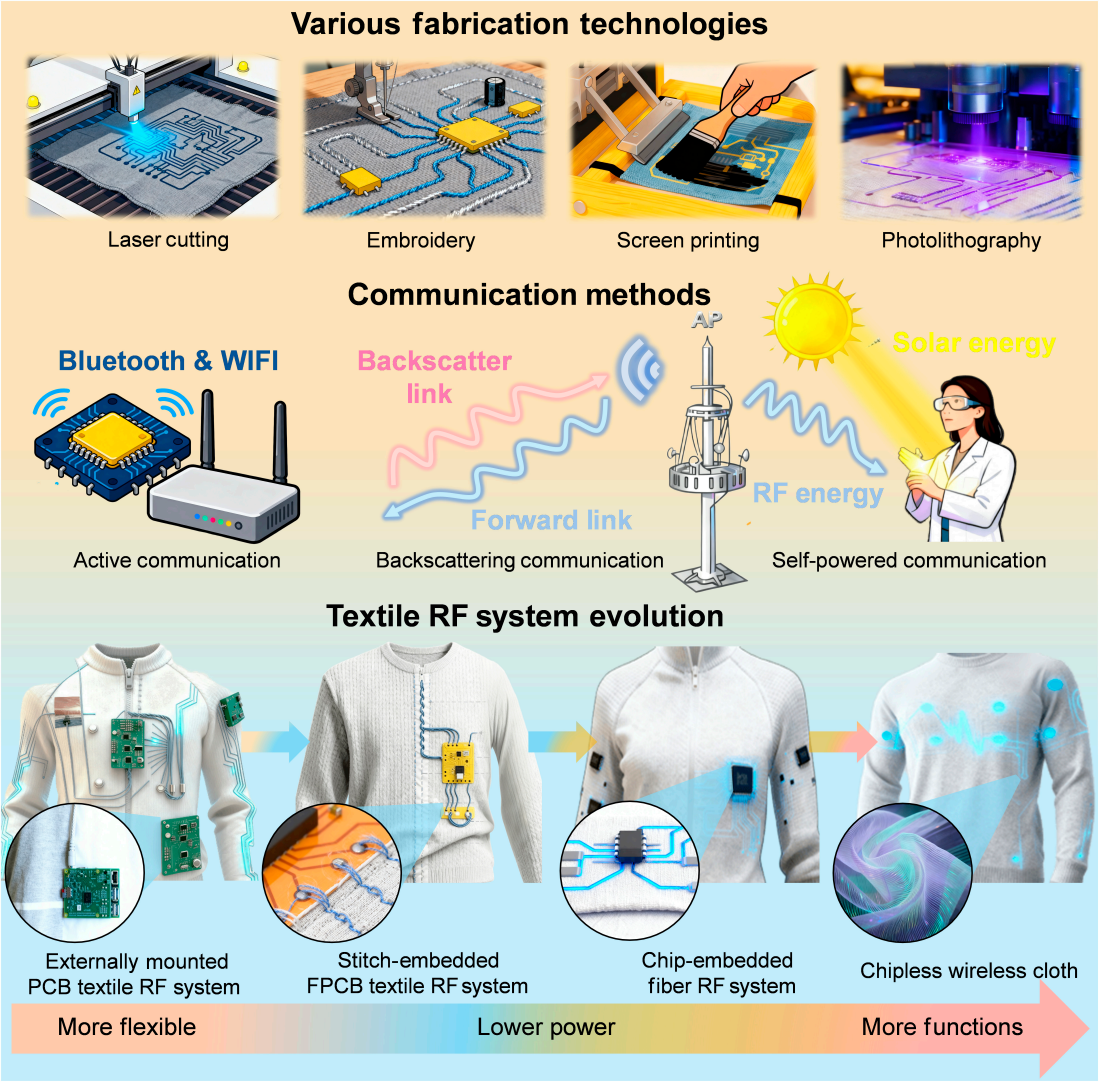
Conclusion and outlook of textile-based RF system across fabrication technologies, communication methods, and system integration.

With the rapid development of flexible and wearable electronics, textile-based RF technologies have gradually evolved from early-stage, locally integrated functional units into highly systematized, multidimensional sensing and distributed intelligent full-textile electronic platforms. This review summarizes the latest advances in textile-based reconfigurable antennas, active metasurfaces, and wearable textile RF systems, systematically outlining the developmental pathway from relatively simple active antenna and metasurface structures, to multi-device, multifunctional system integration, and points toward the future goal of highly flexible, wearable, and potentially chipless full-textile platforms (Fig. 10). Antennas, as fundamental units for signal radiation and reception, provide the physical substrate and signal interface for system functionalities; metasurfaces enable localized control over frequency, polarization, and radiation direction, extending capabilities from single-point devices to region-level intelligent beam manipulation; complex systems further integrate multiple devices, realizing energy self-supply, low-power communication, and environmental sensing, thereby achieving cross-scale integration from local performance optimization to system-level functionality. This evolution not only reflects technological breakthroughs in material design, flexible interconnects, and micro-nano fabrication processes but also highlights the importance of interdisciplinary collaboration. Despite significant recent progress in material development, there are still several key points that need further research when looking forward:

1. Materials: Although conductive fibers and textile-compatible substrates have enabled the integration of RF components into fabrics, challenges remain in balancing electrical performance with mechanical strength and comfort. Conductive yarns often suffer from instability under repeated bending, while low-loss dielectric textiles often exhibit excessively low permittivity, which hinders device miniaturization and RF matching. Future work should focus on designing composite fibers with improved conductivity, mechanical durability, and resistance to sweat and washing. Multifunctional textiles that combine breathability, waterproofing, and EM stability will be key to achieving robust and truly wearable RF platforms that support the evolution from externally mounted to stitch-embedded systems.
2. Fabrication: Existing fabrication strategies, including embroidery, printing, pasting, and photolithography, offer a diverse toolkit with respective advantages for different integration levels, but their resolution and reliability often lag behind those of conventional electronics. Hybrid interconnects and multilayer circuits on porous textiles face issues of long-term adhesion. Looking ahead, scalable high-precision patterning methods and textile-compatible flexible soldering techniques are needed to achieve complex, multilayer RF circuits. Bio-friendly fabrication approaches may further enable seamless integration of electronic functions directly into woven structures.
3. EM design: EM design for textile-based RF systems faces key challenges from mechanical deformation and environmental factors, particularly strain-induced frequency drift and impedance mismatch in low-power applications like backscattering. Future solutions should focus on adaptive impedance networks, topology-optimized structures, and conformal radiation control to maintain operational stability. The integration of reconfigurable elements with AI-assisted EM modeling and digital-twin platforms enables intelligent self-correction under dynamic conditions.
4. System integration: While many demonstrations have achieved energy harvesting, low-power communication, or localized sensing, the integration of all these functions into a lightweight, wearable, and long-duration textile system remains challenging. Future directions include co-optimized architectures that unify multi-communication, computation, and multi-information sensing within the textile, leveraging highly efficient multi-source energy harvesting. This will enable advanced low-power or even self-sustained wearable RF systems, comparable to and potentially surpassing current smartwatches and fitness bands. Ultimately, autonomous textile RF systems could serve as intelligent interfaces for health monitoring, human–machine interaction, and pervasive IoT applications.

## Data Availability

No new data were generated or analyzed in this study.
